# Case report: A rare case of isolated sigmoid Rosai-Dorfman disease on contrast-enhanced CT and ^18^F-FDG PET/CT

**DOI:** 10.3389/fmed.2024.1425112

**Published:** 2024-06-12

**Authors:** Wanling Qi, Zhehuang Luo, Mingyan Shao, Min Chen, Fengxiang Liao, Linfeng Hu

**Affiliations:** ^1^Department of Nuclear Medicine, Jiangxi Provincial People’s Hospital, The First Affiliated Hospital of Nanchang Medical College, Nanchang, China; ^2^Department of Pathology, Jiangxi Provincial People’s Hospital, The First Affiliated Hospital of Nanchang Medical College, Nanchang, China; ^3^Department of Pediatrics, Jiangxi Provincial People’s Hospital, The First Affiliated Hospital of Nanchang Medical College, Nanchang, China

**Keywords:** Rosai-Dorfman disease, histiocytosis, CT, ^18^F-FDG, PET/CT

## Abstract

Rosai-Dorfman disease (RDD) is an uncommon histiocytic disorder that occurs in nodal and/or extranodal sites. Extranodal RDD exhibits a wide range of clinical and radiological presentations, frequently leading to misdiagnoses. Involvement of the gastrointestinal (GI) system is uncommon, accounting for less than 1% of the reported cases. Here we present a case of a 54-year-old male who complained of abdominal distention and was diagnosed with RDD affecting the sigmoid colon, manifesting as a sigmoid mass. The patient had a past medical history of liver transplantation due to hepatocellular carcinoma (HC). This report details the multiphase contrast-enhanced computed tomography (CT) and fluorodeoxyglucose (^18^F-FDG) positron emission tomography (PET-CT) imaging findings of RDD involving the sigmoid colon without lymphadenopathy, and a review of the relevant literature is provided.

## Introduction

Rosai-Dorfman disease (RDD), initially described by Pierre Paul Louis Lucien Destombes in 1965 (1), was later characterized pathologically by Juan Rosai and Ronald Dorfman in 1969 (2), has the potential to affect any tissue or organ. Approximately 25–43% of patients present with extranodal involvement, including the skin, orbits, bones, upper respiratory tract, gastrointestinal (GI) system, genitourinary system, central nervous system, and endocrine glands (3). Although extranodal disease frequently occurs with nodal involvement, isolated extranodal disease is uncommon. This report documents a case of a 54-year-old male with RDD confined to the sigmoid colon. We discuss the imaging findings and diagnosis from multi-phase contrast-enhanced computed tomography (CT) and fluorodeoxyglucose (^18^F-FDG) positron emission tomography (PET-CT), as well as the patient’s treatment and follow-up. While RDD can involve the GI system, such cases are still infrequent in the literature (4–9). To the best of our knowledge, this is the first report of RDD affecting the sigmoid colon, and the utility of PET/CT in such a case has not been previously described.

## Case description

A 54-year-old male patient presented with abdominal fullness for 20 days before admission. He denied symptoms of fever, nausea, vomiting, hematemesis, melena, weight loss, night sweats, and lymph node enlargement. Moreover, the patient underwent a liver transplantation eight years ago due to hepatocellular carcinoma (HC) and has since made a good recovery.

**Figure 1:**
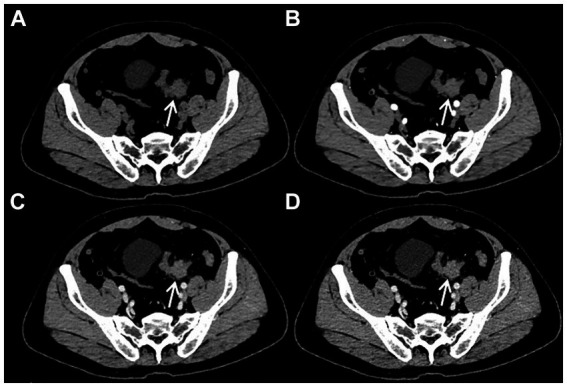
Plain CT imaging [**(A)**, arrow ↑] revealed a round soft tissue density lesion in the left lower quadrant abdomen, with the boundary of the lesion with the sigmoid colon not being distinct. On multiphasic contrast-enhanced CT scans [**(B–D)**, arrow ↑], the lesion demonstrated mild to moderate enhancement. The CT values for the non-contrast scan phase, arterial phase, venous phase, and delayed phase were 46 HU, 57 HU, 67 HU, and 70 HU, respectively.

**Figure 2:**
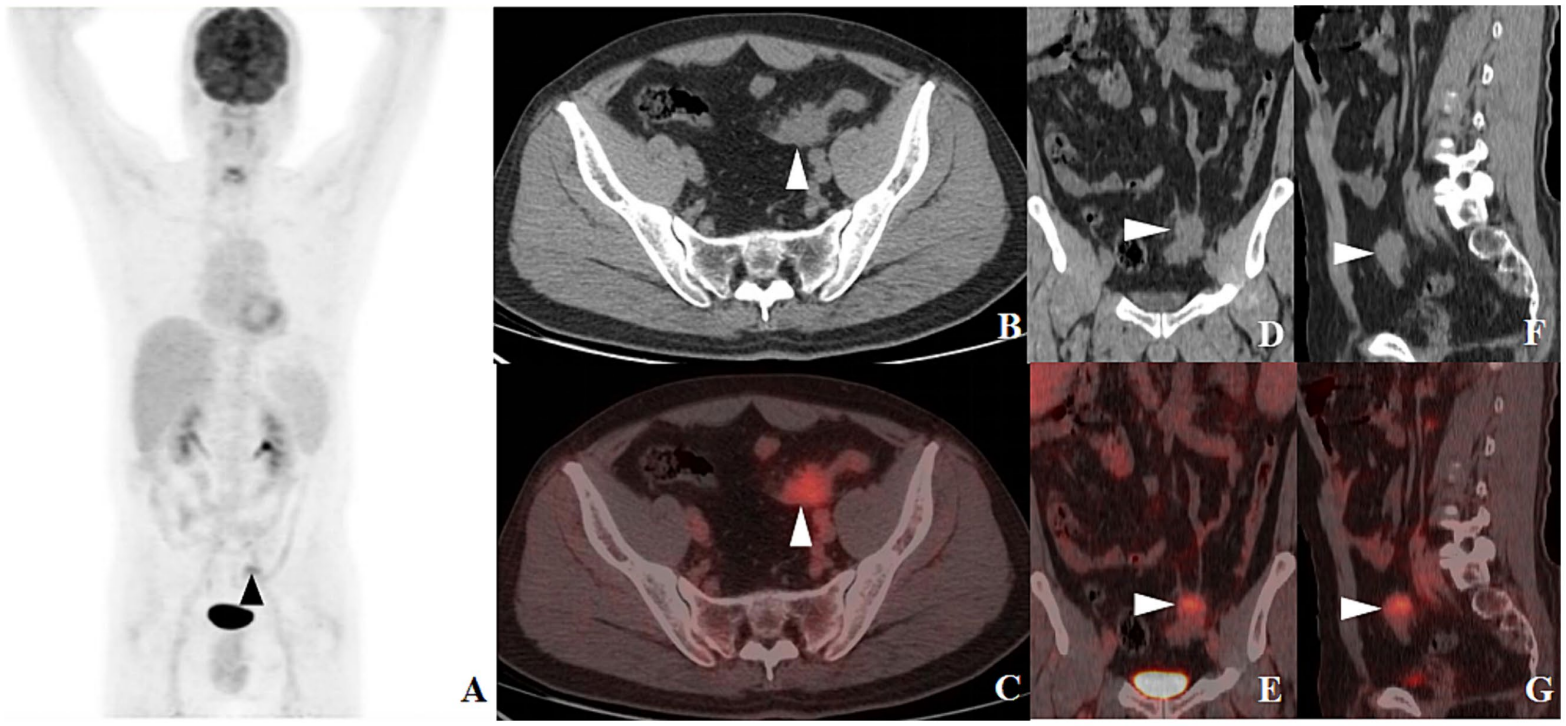
^18^F-FDG PET-CT MIP image [**(A)**, arrow ▲] revealed a lesion with increased FDG uptake in the left lower quadrant of the abdomen. Axial CT [**(B)**, arrow ▲] and fused axial PET-CT [**(C)**, arrow ▲] images depicted an isolated, round soft tissue density mass in the same area, with increased FDG uptake and a SUVmax of 3.6. Coronal and sagittal CT images [**(D,F)**, arrow ▲] and their corresponding fused PET-CT counterparts [**(E,G)**, arrow ▲] revealed an indistinct boundary between the mass and the sigmoid colon.

After admission, the patient underwent a series of related laboratory tests, a multiphase contrast-enhanced CT, and an ^18^F-FDG PET/CT. The results of the blood routine tests showed that white blood cell count, red blood cell count, and C-reactive protein levels were all within normal limits. Tumor markers such as carcinoembryonic antigen (CEA), alpha-fetoprotein (AFP), and cancer antigen 19–9 (CA19-9) were within normal ranges. The plain CT scan image (Figure 1A) identified a nodular mass with isodensity in the left lower quadrant of the abdomen, measuring approximately 2.3 × 3.1 cm, with an indistinct border in relation to the sigmoid colon. No enlarged lymph nodes were observed in the surrounding fat spaces or in other areas of the body. The multiphase contrast-enhanced CT scan images (Figures 1B–D) demonstrated the lesion exhibiting mild to moderate enhancement. The CT values recorded for each phase are as follows: non-contrast scan phase 46 HU, arterial phase 57 HU, venous phase 67 HU, and delayed phase P70 HU. Based on these findings, the preliminary CT interpretation favored an inflammatory lesion, with a recommendation for follow-up after antibiotic therapy. Given the patient’s history of HC, an PET-CT scan was performed to rule out the possibility of metastasis. The maximum intensity projection (MIP) image (Figure 2A) revealed a focal area of increased FDG uptake in the left lower quadrant of the abdomen. The axial CT and fused axial PET-CT images (Figures 2B,C) revealed an isolated, round, soft-tissue mass with isodensity in the left lower quadrant, showing increased FDG uptake and a maximum standardized uptake value (SUVmax) of 3.6. The corresponding coronal and sagittal CT and fused PET-CT images (Figures 2D–G) demonstrated that the mass had an indistinct margin in relation to the sigmoid colon, suggesting a gastrointestinal stromal tumor (GIST) as a likely diagnosis. The whole-body PET/CT showed the absence of any FDG avid visible lymph node or FDG avid visible disease elsewhere in the regions of the body surveyed.

**Figure 3:**
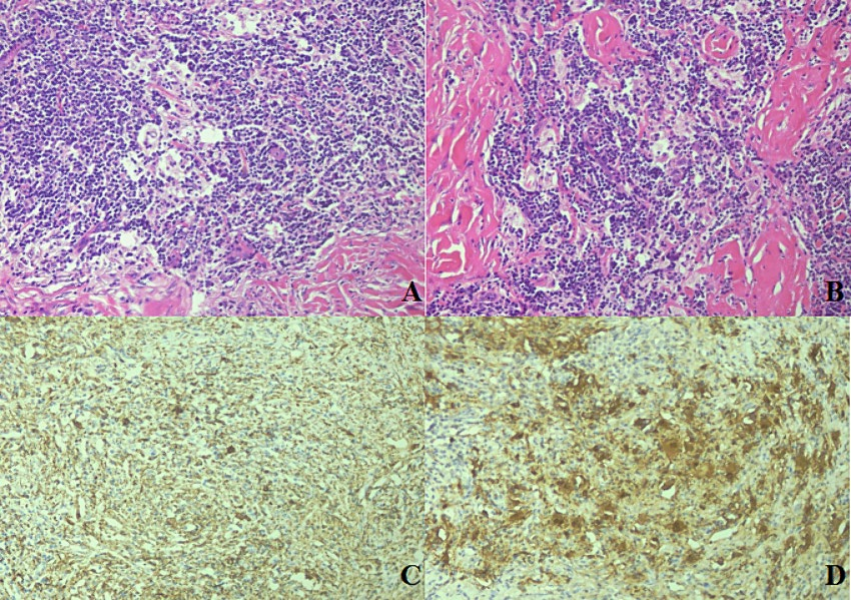
Histological and immunohistochemical characteristics of sigmoid RDD. The histological section exhibited clusters of histiocytes amidst lymphocytes and plasmacytes, with a fibrotic background. Emperipolesis, characterized by small lymphocytes and plasmacytes within the cytoplasm of the histiocytes, was evident [**(A,B)**; HE 20×]. The large histiocytes were positive for CD68 [**(C)**; HE 40×] and S100 [**(D)**; HE 40×].

To further investigate the possibility of the lesion originating from the intestinal tract, a sigmoidal-rectal endoscopic ultrasonography was conducted, which yielded no evidence of abnormality. Experiencing abdominal discomfort and harboring significant concern over the potential for malignancy, the patient underwent laparoscopic resection to obtain a definitive diagnosis. Intraoperative visualization indicated a smooth intestinal mucosa with an ill-defined, tough, off-white area in the serosal layer. Microscopic examination disclosed aggregates of obese histiocytes and plasma cells within the serosal and muscularis propria layers, with lymphocytes present in focal clusters. Peripheral collagenous fibers were observed in a braided, bundled arrangement. The presence of small lymphocytes and plasma cells within the cytoplasm of some histiocytes, a phenomenon known as emperipolesis, was noted (Figures 3A,B). Immunohistochemical analysis confirmed that the histiocytes were positive for CD68 (Figure 3C), S100 (Figure 3D), and CD163, while negative for CK, ALK, CD1a, and Langerin. The pathological findings were consistent with a diagnosis of Rosai-Dorfman disease affecting the serosal surface and deep muscular layer of sigmoid colon. A year post-surgery, routine follow-up examinations have revealed no signs of recurrence.

## Discussion

Rosai-Dorfman disease (RDD) is currently regarded as a neoplastic condition characterized by the clonal expansion of histiocytes in lymph nodes and/or extranodal sites (10). It is noteworthy that RDD is a rare disorder, with an estimated prevalence of 1 in 200,000 and an annual incidence of approximately 100 cases (11). The precise etiology of RDD remains elusive, but proposed factors include viral infections, such as Epstein–Barr virus and other human herpesviruses, autoimmune dysregulation, genetic mutations, and disruptions in signaling pathways (11–17). Being widely heterogeneous and presenting a variety of clinical manifestations, RDD can range from isolated cases to those associated with other diseases such as autoimmune, hereditary, malignant or IgG4 (18, 19). The GI system is one of the least commonly affected sites. It predominantly affects middle-aged females, presenting with a range of non-specific symptoms that include abdominal pain, diarrhea, rectal bleeding, and weight loss (7–9). This report details a case of RDD confined to the sigmoid colon in a 54-year-old male patient, who presented with abdominal distention. Given that neoplasia-associated RDD refers to RDD that occurs either precedes or arises subsequent to lymphoma or myelodysplastic syndrome (20), the present case, despite having a history of HC, still falls under the category of an isolated form of RDD.

Histopathologically, RDD lesions are distinguished by the infiltration of large histiocytes within the tissue, set against a backdrop of mixed inflammation. These extranodal lesions resemble nodal RDD but tend to exhibit increased fibrosis, sclerosis, a lower density of histiocytes, and less pronounced emperipolesis (21). A panel of immunostains is typically employed to confirm the diagnosis, with positive markers including S100, CD68, and CD163, and negative markers such as CD1a, ALK, and langerin (22).

The radiological characteristics of extranodal RDD vary depending on the organ involved. On plain CT scans, extranodal RDD typically presents as well-demarcated, single or multiple lesions with isodense or hyperdense masses. Multiphasic contrast-enhanced CT often reveals significant and progressive homogeneous enhancement (20, 23, 24). MRI findings include lesions that are predominantly hypointense to isointense on T1-weighted (T1WI) images and display variable signal intensity on T2-weighted (T2WI) images, which may correspond to the presence of free radicals generated by macrophages during active phagocytosis (23, 25, 26). As most RDD-involved sites are FDG-avid, ^18^F-FDG PET/CT could identify lesions that were not visible on conventional examinations, which has higher sensitivity and lower false-positive rates (27). The FDG avidity in RDD lesions is due to the high glucose metabolism of the proliferating histiocytes (28). A decrease in FDG uptake can signify effective treatment or spontaneous regression of RDD (27). Thus, the ^18^F-FDG PET/CT imaging is a whole-body examination that, despite lacking specificity for the diagnosis of RDD, plays a crucial role in assessing disease extent, providing a comprehensive staging of the condition, guiding the biopsy, and monitoring treatment efficacy, considering that RDD can occur in any tissue or organ. In this case, the contrast-enhanced CT scan revealed the lesion with mild to moderate enhancement, and the PET/CT scan displayed correspondingly mild to moderate FDG uptake. Both the intensity of enhancement and the SUVmax values were lower than those reported in previous literature (20, 27, 28). These findings could be related to the proportional composition of the histiocytes, lymphocytes, and fibrosis within the lesion.

RDD in the ileum and colon can be difficult to distinguish from other intestinal pathologies such as Crohn’s disease, ulcerative colitis, and colon cancer. GI RDD tends to present with more substantial intestinal wall thickening than Crohn’s disease and ulcerative colitis, and it may be accompanied by intestinal obstruction (7). Endoscopic ultrasound-guided fine needle aspiration and cytological examination can provide a qualitative diagnosis (29). In this case, due to the patient’s history of HC, it was necessary to differentiate from metastasis. However, the occurrence of HC spreading to the sigmoid colon was uncommon, and the lesion in this region lacked significant enhancement. Furthermore, the patient’s AFP levels were normal. Taking all these aspects into account, metastasis was not suspected in the diagnosis. Since the sigmoid mass only invaded the serosal layer and muscularis propria layer of the intestine, endoscopic ultrasonography failed to detect any obvious abnormalities. Generally, GI RDD poses diagnostic challenges and requires a multi-modal approach to identification.

The treatment of RDD remains without a standardized protocol, with diverse management strategies. In general, observation is appropriate for patients with uncomplicated lymphadenopathy and asymptomatic cutaneous lesions. Surgical excision is often curative for patients with solitary extranodal disease (30). Systemic therapies are typically employed for multifocal or treatment-resistant cases (11, 31). Therefore, timely and intuitive assessment of the treatment efficacy through PET/CT is critical for determining effective options when selecting new treatments (27). In this case, the isolated lesion in the sigmoid colon was surgically removed, and the patient’s prognosis was positive.

## Conclusion

In conclusion, we report a rare case of unifocal extranodal RDD occurring in the sigmoid. CT imaging showed a round, ill-defined isodense mass with mild to moderate enhancement on multiphasic contrast-enhanced scans. Simultaneous PET/CT imaging showed mild to moderate FDG uptake. Given the FDG-avid nature of RDD lesions, PET/CT offers advantages over conventional imaging modalities for initial assessment, treatment strategy adjustment, and efficacy evaluation. Recognizing the imaging characteristics of RDD necessitates the accumulation of more case studies and the synthesis of clinical experience. The ultimate diagnosis of RDD still depends on biopsy or immunohistochemical analysis.

## Data availability statement

The original contributions presented in the study are included in the article/supplementary material, further inquiries can be directed to the corresponding authors.

## Ethics statement

The studies involving humans were approved by Jiangxi Provincial People’s Hospital Medical Ethics Committee. The studies were conducted in accordance with the local legislation and institutional requirements. Written informed consent for participation in this study was provided by the participants’ legal guardians/next of kin. Written informed consent was obtained from the individual(s) for the publication of any potentially identifiable images or data included in this article.

## Author contributions

WQ: Writing – review & editing, Writing – original draft, Methodology. ZL: Writing – original draft, Formal analysis. MS: Writing – review & editing, Data curation. MC: Writing – review & editing, Investigation. FL: Writing – review & editing, Methodology. LH: Writing – review & editing, Data curation.
